# The Impact of Combined Written and Audio-Visual Information on Preoperative Anxiety Among Adult Undergoing Total Knee Replacement Surgery: A Quasi-Experimental Study

**DOI:** 10.3390/healthcare14213779

**Published:** 2026-11-05

**Authors:** Waleed Sarhan, Fuad H. Abuadas, Musab Abdallah, Wisal Al Sheikh Abdallah, Ahmad Al Baker, Katherine Mae Nualda, Noor Ab Rahman

**Affiliations:** 1 Department of Nursing, Sultan Bin Abdulaziz Humanitarian City, Riyadh 13571, Saudi Arabia; wsarhan101@gmail.com (W.S.); muaabdallah@outlook.com (M.A.); wisalahmed2014@gmail.com (W.A.S.A.); ah_baker@hotmail.com (A.A.B.); kathcee08@yahoo.com (K.M.N.); noorhairanie3001@gmail.com (N.A.R.); 2 Primary Care Nursing Department, Faculty of Nursing, Al-Ahliyya Amman University, Amman 19328, Jordan

**Keywords:** anxiety, total knee replacement surgery, preoperative, combined written and audio-visual education

## Abstract

**Background/Objectives:** This study aimed to evaluate the effect of combined written and audio-visual preoperative education on reducing preoperative anxiety and information desire among adult patients undergoing total knee replacement surgery. **Methods:** A quasi-experimental model was used. The study was conducted in adult surgical wards of a Sultan Bin Abdul-Aziz Humanitarian City (SBAHC). One hundred thirty-six patients voluntarily participated in the study, 68 of them were in the Control group (CG) and 68 of them were in the Experimental Group (EG). While routine oral education only was given to CG, a combined written and audio-visual preoperative education was also provided to EG. Data were collected using a structured self-administered questionnaire (Arabic version of Amsterdam Preoperative Anxiety and Information Scale (APAIS)). The patients in both groups filled in the questionnaire and APAIS preoperatively at two time points before and after the intervention. **Results:** At baseline, the mean anxiety score was 12.90 ± 2.26 in the control group and 13.10 ± 1.86 in the experimental group (*t* = −0.58, *p* = 0.56). Similarly, the mean information desire score was 6.84 ± 1.43 in the control group and 7.16 ± 1.59 in the experimental group (*t* = −1.25, *p* = 0.21), indicating that the two groups were comparable prior to the intervention. After the intervention, the experimental group demonstrated significantly lower anxiety levels (9.26 ± 1.84) compared with the control group (11.49 ± 1.65) (*t* = 7.40, *p* < 0.001). Similarly, the information desire score was significantly lower in the experimental group (2.31 ± 0.58) than in the control group (5.46 ± 1.01) (*t* = 22.22, *p* < 0.001). **Conclusions:** A combined written and audio-visual preoperative education decreased preoperative anxiety and information desire among adult patients undergoing total knee replacement surgery.

## 1. Introduction

Total knee replacement (TKR) is an established intervention for advanced knee joint disease, but its anticipated benefits are preceded by a demanding perioperative pathway involving anesthesia, major surgery, postoperative pain, early mobilization, rehabilitation, and temporary loss of independence [1,2]. Uncertainty about these experiences may elicit preoperative anxiety, a situation-specific response characterized by apprehension, tension, and fear related to surgery and anesthesia [3]. This response is clinically relevant because it may affect physiological stability, anesthetic and analgesic requirements, pain perception, and recovery [4,5,6]. Within total knee replacement populations specifically, greater preoperative anxiety has been associated with more severe postoperative pain and poorer early joint function [2,7]. Preoperative anxiety is therefore a potentially modifiable perioperative factor and an important target for patient-centered nursing care [4,5,8].

The reported burden of preoperative anxiety varies widely because studies differ in surgical populations, cultural settings, assessment instruments, and thresholds [4,5,9]. A systematic review and meta-analysis of patients in low- and middle-income countries estimated that approximately one in two surgical patients experienced preoperative anxiety, but substantial between-study heterogeneity limited generalization across procedures and settings [5]. Individual studies likewise reported markedly different estimates, including 22.9% among elective surgical patients in Nepal, 53.6% in an Ethiopian hospital, and 70.3% in another Ethiopian surgical population [10,11]. In a Palestinian hospital sample assessed with the Amsterdam Preoperative Anxiety and Information Scale (APAIS), 27.1% had high anxiety and 57.1% reported a high need for information [9]. These differences indicate that prevalence estimates from mixed surgical samples should not be assumed to represent adults undergoing TKR in a specific healthcare context [4,5,9].

Preoperative education is a plausible non-pharmacological strategy because unfamiliarity with the perioperative pathway and inadequate understanding of care can intensify uncertainty and anxiety [3,12]. Routine verbal counseling permits clarification and interpersonal support, but its effectiveness may vary according to consultation time, consistency of delivery, and patients’ ability to recall information when anxious [13,14]. Written information provides a stable resource for later review, whereas audio-visual information can illustrate the hospital journey, anesthesia, surgery, and recovery in a standardized and concrete form [12,15]. Combining written and audio-visual materials may reinforce key information and allow patients and family members to review the educational content repeatedly [12,15].

Although preoperative educational interventions appear promising, the available evidence does not identify a consistently optimal delivery format for patients undergoing total knee replacement [1,8,13]. Many studies have involved heterogeneous surgical samples or procedure-specific populations whose concerns and recovery pathways differ from those associated with joint replacement [14,15,16]. Interventions have ranged from electronic text and conventional video to head-mounted virtual reality, with inconsistent comparators, outcome instruments, and assessment intervals [8,13,14,17]. Immersive technologies may also require equipment, staff support, and implementation resources that constrain routine use [8,13].

Several existing trials have evaluated either a single educational medium or a technologically intensive intervention rather than a pragmatic package integrating written and audio-visual information with routine clinical education [13,14,15]. The transferability of these findings to Arabic-speaking adults undergoing elective total knee replacement remains uncertain because much of the available evidence comes from other procedures, populations, and educational formats. Regional studies nevertheless indicate substantial anxiety and information needs, and a validated Arabic assessment instrument is available [9,18]. Consequently, uncertainty remains about whether a brief, repeatable, multimodal educational package can reduce anxiety and address information needs in this population [9,12,18]. Evaluating such an approach may support more consistent nursing education and better identification of patients requiring additional preparation [3,19].

Previous studies have examined multimedia and virtual reality education as approaches to addressing preoperative anxiety and information needs [13,15]. Combined written and audio-visual information may help patients understand unfamiliar aspects of anesthesia, surgery, and recovery. Audio-visual materials can illustrate the anticipated perioperative experience, while written materials provide a reference for subsequent review. These complementary formats may reinforce understanding, reduce uncertainty, and increase perceived preparedness, potentially reducing anxiety. This rationale is also consistent with the reported association between preoperative anxiety and the desire for additional information [20,21]. However, these mechanisms remain proposed explanations, as understanding, uncertainty, and preparedness were not directly measured in this study. Therefore, the purpose of this study was to evaluate the effect of combined written and audio-visual preoperative education on reducing preoperative anxiety and information desire among adult patients undergoing total knee replacement surgery. The study examined two directional hypotheses: patients receiving combined written and audio-visual education would have lower post-intervention APAIS anxiety scores (H1) and lower information desire scores (H2) than those receiving routine oral education.

## 2. Materials and Methods

### 2.1. Design

This study used a quasi-experimental, two group pre-test–post-test design to examine the effects of combined written and audio-visual information on anxiety between patients having total knee replacement surgery at Sultan Bin Abdulaziz Humanitarian City (SBAHC). Data collection occurred in the adult surgical wards, between 27 July 2025 and 16 January 2026.

### 2.2. Participants

The population was adult patients a few days before elective total knee replacement surgery. The G*Power software (version 3.1.9.7; Heinrich Heine University Düsseldorf, Düsseldorf, Germany). was used to determine sample size. The minimum sample size required was 128 patients based on a statistical power of0.80, a level of 0.05 and medium effect size (d = 0.5). Additional participants were added at recruitment to allow for incomplete responses and attrition. One hundred fifty-five patients were approached for the study, 136 completed all measures needed in order to be included in the final analyses with a response rate of 87.7%. Nineteen patients were excluded due to incompletion of one or more measures (9 were from the control group and 10 were from the experimental group). Finally, the dataset included 68 from the experimental and 68 from the control group. Patients aged 18 years or above, had an indication for elective total knee replacement (TKR), and could understand Arabic were included. Patients were excluded if they were scheduled for day surgery, or undergoing a surgical procedure other than total knee replacement.

### 2.3. Sampling

Eligible patients were selected after completion of the preoperative clinic process using a convenience sampling method from the surgical information center. Participants were allocated to groups using a rolling enrollment method [22]. In this method, the first participant agreeing to take part was allocated to the experimental group, the second participant to the control group, and an alternating sequence continued for each subsequent participant on a daily basis across data collection until participants reached minimum sample size.

### 2.4. Data Collection

**Figure 1:**
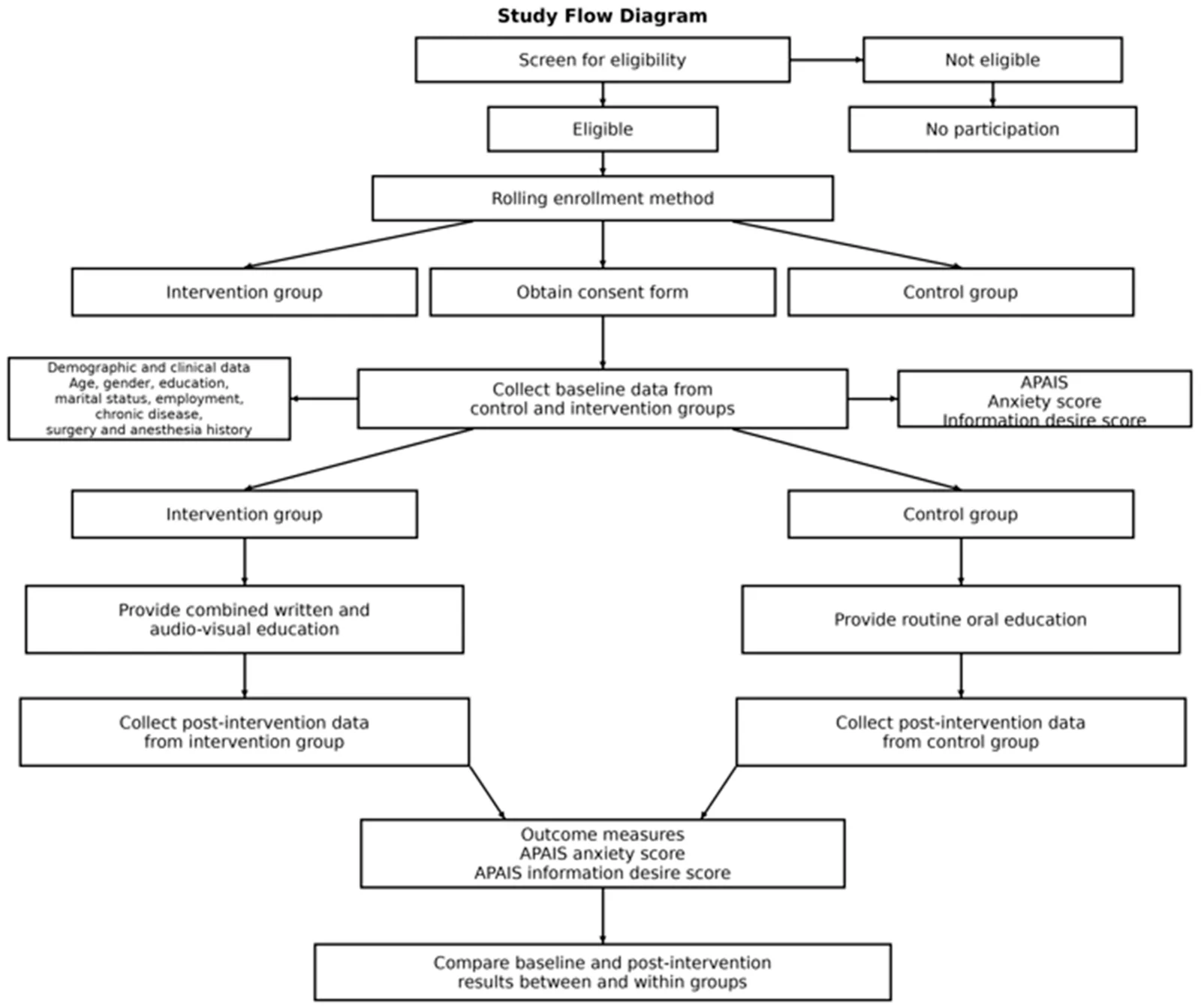
Study flow diagram.

Following confirmation of eligibility and obtaining informed consent, baseline data collection was performed in both groups. Baseline APAIS was administered at the preoperative clinic before education. The experimental group received the combined written and audio-visual education at the surgical information center before the day of surgery. The control group received routine oral education upon hospital admission. Post-intervention APAIS was administered to both groups upon admission for surgery and before surgery. Thus, the post-test was conducted at the same perioperative time point in both groups, although the interval between education and post-test was longer in the experimental group. Education was provided by the case manager, who described the materials to the patient and family members, and then electronically sent it through email and WhatsApp for later review. Figure 1 shows the flow of participants throughout the study and includes screening, enrollment, group allocation, assessment at baseline and post-intervention as well as intervention delivery.

### 2.5. Intervention

The written and audio-visual educational materials were developed by the study researchers for adults undergoing total knee replacement. The draft materials were reviewed by two experts: an orthopedic surgeon with experience in total knee replacement and a perioperative nurse with experience in preoperative patient education. They assessed the materials for clinical accuracy, relevance, and clarity, and their feedback was considered in finalizing the materials. The case manager introduced the materials to participants and their family members and sent electronic copies by email or WhatsApp for later review. The educational package for the experimental group included information about treatment team members and their roles, preoperative preparation, home preparation post discharges, an overview of surgical procedure and anesthesia type and recovery period. The duration of the audio-visual session was approximately 15 min.

### 2.6. Instruments

Data were collected using a structured self-administered questionnaire and the Arabic version of Amsterdam Preoperative Anxiety and Information Scale (APAIS) [23]. The demographic and clinical questionnaire captured gender, age, educational level, marital status, employment status, history of previous surgery, history of previous anesthesia, presence of chronic disease, and use of anxiety-related medication. Preoperative anxiety and desire for information were assessed using the APAIS, a brief self-report instrument developed specifically for surgical patients [23]. The scale contains six items rated on a 5-point Likert scale ranging from 1, not at all, to 5, extremely. Four items, namely items 1, 2, 4, and 5, are summed to generate the anxiety subscale, which ranges from 4 to 20. The remaining two items, items 3 and 6, are summed to generate the information desire subscale, which ranges from 2 to 10. An anxiety score of 11 or higher indicates clinically relevant preoperative anxiety. For the information desire subscale, scores of 5 to 7 reflect an average need for information, whereas scores of 8 to 10 indicate a high information requirement. The Arabic version of APAIS has demonstrated good validity and reliability in Arabic-speaking surgical patients. ALMesned et al. [18] reported high internal consistency, with Cronbach’s alpha values of 0.851 for the anxiety subscale and 0.827 for the information subscale, and confirmed the original two-factor structure of the scale. In the present sample (N = 136), internal consistency was acceptable for the anxiety subscale (Cronbach’s α = 0.701) and good for the information desire subscale (Cronbach’s α = 0.803).

### 2.7. Statistical Analysis

Data were analyzed utilizing the Statistical Package for the Social Sciences, version 26. Descriptive statistics were used to summarize participant characteristics. Continuous variables were expressed as means and standard deviations, whereas categorical variables were presented as frequencies and percentages. Chi-square test (χ^2^) was used to compare categorical demographic and clinical variables at baseline. Anxiety-related medication use was compared using a two-sided Fisher’s exact test because of small expected cell counts. Post-intervention APAIS anxiety and information desire scores were compared between groups using separate analyses of covariance (ANCOVA), adjusting for the corresponding baseline score. Adjusted means and between-group mean differences were reported with 95% confidence intervals. HC3 heteroskedasticity-robust standard errors were used to calculate confidence intervals and *p*-values for both outcomes. Partial eta-squared (η^2^), calculated from conventional ANCOVA sums of squares, was reported as an effect-size measure. Statistical significance was set at *p* < 0.05. Normality and relevant statistical assumptions were checked before conducting inferential analyses.

### 2.8. Ethical Considerations

This study was conducted after obtaining ethical approval from the institutional review board committee at Sultan Bin Abdulaziz Humanitarian City (SBAHC), in accordance with the principles of the Declaration of Helsinki [24,25]. The study received approval number 137-2025-IRB (06 February 2025). All eligible patients were informed of the study’s purpose, the voluntary nature of participation, and their right to withdraw at any time without affecting the care they received prior to participating. Written informed consent was obtained from all participants before data collection. To maintain confidentiality and anonymity, each participant was assigned a unique code number, and no identifying personal information was included in the dataset. All collected data were stored securely in password-protected electronic files and were reported in aggregate form only.

## 3. Results

One hundred fifty-five participants were approached in Sultan Bin Abdulaziz Humanitarian City (surgical unit) from 27 July 2025 to 16 January 2026. One hundred thirty-six participants (about an 87.7% response rate) completed the pre-test questionnaires, health education intervention, and post-test questionnaires. Nineteen participants were excluded because they did not complete one or more of these measures.

### 3.1. Sample Socio-Demographic Characteristics

At baseline, the sample consisted of 68 participants in the experimental group and 68 in the control group. The mean age was similar in both groups, 48.35 ± 4.43 years in the experimental group and 48.56 ± 4.50 years in the control group, with no significant difference (*t* = 0.27, *p* = 0.79). Females represented the majority in both groups, 57.4% in the experimental group and 66.2% in the control group, with no significant difference (χ^2^ = 1.12, *p* = 0.29). Most participants were married, 70.6% in the experimental group and 63.2% in the control group (χ^2^ = 0.99, *p* = 0.61). Secondary education was the most common educational level in both groups, and the difference between groups was not statistically significant (χ^2^ = 5.84, *p* = 0.06). The majority of participants were not employed, 88.2% in the experimental group and 89.7% in the control group (χ^2^ = 0.08, *p* = 0.78). A high proportion of participants reported chronic diseases, 79.4% in the experimental group and 64.7% in the control group (χ^2^ = 3.65, *p* = 0.06). Previous surgical history and history of anesthesia were also similar between groups, with no significant differences (*p* = 0.08 and *p* = 0.21, respectively). Anxiety-related medication use was low and did not differ significantly between groups (Fisher’s exact test, *p* = 0.208). No statistically significant baseline differences were detected. Details of the socio-demographic characteristics are displayed in Table 1.

### 3.2. Baseline Preoperative Anxiety and Information Desire Scores (Before Intervention)

At baseline, participants reported varying levels of preoperative anxiety and information needs as measured by the Arabic version of APAIS. As shown in Table 2, responses to the individual APAIS items indicated that a considerable proportion of participants experienced moderate to high levels of concern related to both anesthesia and the surgical procedure. Specifically, 33.1% of participants reported moderate worry about the anesthetic, while 29.4% and 12.5% reported high and extreme levels of worry, respectively. Similarly, 37.5% indicated that anesthesia was moderately on their mind, whereas 27.2% and 15.4% reported high and extreme levels. Regarding the need for information about anesthesia, 32.4% of participants reported a moderate desire for information, 34.6% reported a high desire, and 17.6% reported an extreme desire for additional information.

Procedure-related concerns were also evident. As presented in Table 2, 39.0% of participants reported moderate worry about the procedure, while 25.0% and 14.7% reported high and extreme worry, respectively. In addition, 38.2% stated that the procedure was moderately on their mind, and 31.6% reported that it was on their mind to a high degree. Regarding information needs about the procedure, 44.1% of participants reported a moderate desire for information, while 25.0% and 19.9% reported high and extreme levels of information need.

The APAIS scoring system includes two subscales: an anxiety score derived from items 1, 2, 4, and 5 (range 4–20) and an information desire score derived from items 3 and 6 (range 2–10). A cut-off score of 11 or higher on the anxiety subscale indicates clinically relevant preoperative anxiety, while information scores of 5–7 indicate an average need for information and 8–10 indicate a high information requirement.

As shown in Table 3, the mean anxiety score was 12.90 ± 2.26 in the control group and 13.10 ± 1.86 in the experimental group. Both values exceed the APAIS cut-off point of 11, indicating that participants in both groups were experiencing clinically relevant preoperative anxiety prior to the intervention. The mean information desire score was 6.84 ± 1.43 in the control group and 7.16 ± 1.59 in the experimental group, which falls within the APAIS range for an average information requirement. Independent samples t-tests showed no statistically significant differences between the control and experimental groups for anxiety score (*t* = −0.58, *p* = 0.56) or information desire score (*t* = −1.25, *p* = 0.21), indicating that the two groups were comparable at baseline before the intervention.

### 3.3. Preoperative Anxiety and Information Desire Scores (After Intervention)

Table 4 presents the baseline and post-intervention APAIS anxiety and information desire scores for both groups, together with comparisons adjusted for the corresponding baseline scores using ANCOVA. The adjusted post-intervention anxiety score was significantly lower in the experimental group than in the control group (9.25 vs. 11.50), with an adjusted mean difference of −2.25 points (95% CI: −2.84 to −1.66; *p* < 0.001; partial η^2^ = 0.301). Similarly, the adjusted information desire score was significantly lower in the experimental group (2.31 vs. 5.45), with an adjusted mean difference of −3.14 points (95% CI: −3.42 to −2.87; *p* < 0.001; partial η^2^ = 0.784). Confidence intervals and *p*-values were calculated using HC3 heteroskedasticity-robust standard errors. These findings indicate that the educational intervention was associated with lower post-intervention anxiety and information desire scores after adjustment for baseline scores. The anxiety model explained 31.0% of post-intervention variance (R^2^ = 0.310; adjusted R^2^ = 0.300). The information desire model explained 78.7% (R^2^ = 0.787; adjusted R^2^ = 0.783).

## 4. Discussion

The present study found that adults undergoing total knee replacement surgery experienced clinically relevant preoperative anxiety before the intervention, as baseline APAIS [23] anxiety scores in both groups were above the commonly used clinical cut-off of 11. Both groups were comparable at baseline in their sociodemographic characteristics, anxiety scores, and information desire scores, which suggests that the post-intervention differences were less likely to be explained by pre-existing group imbalance. After the intervention, participants who received the combined written and audio-visual information showed substantially lower anxiety and information desire scores than those in the control group. Moreover, the mean post-intervention anxiety score in the experimental group dropped below the APAIS cut-off, whereas the control group showed only a modest decline, which may indicate that the combined educational intervention had a clinically meaningful effect on psychological preparation before surgery.

While the current study confirms a significant attenuation of anxiety levels following the combined educational intervention, similar results have been observed in other surgical contexts. For instance, Bozkurt, Erkoc, Can, Danıs, and Canat [12] demonstrated that patients undergoing percutaneous nephrolithotomy (PCNL) exhibited a statistically significant decrease in State-Trait Anxiety Inventory (STAI-S) scores (*p* < 0.01) when provided with animated video briefings, whereas the control group receiving only standard information showed no such improvement (*p* = 0.86). These results apparently confirm that structured, multimodal information delivery is a potent non-pharmacological anxiolytic [12]. In the present study, anxiety and information desire both declined after the intervention, especially in the experimental group. This pattern may indicate that as patients became better informed about anesthesia, surgery, and the perioperative process, their fear of the unknown decreased. This interpretation is supported by Jiwanmall et al. [21], who found that the need for additional information regarding surgery and anesthesia was significantly associated with preoperative anxiety, and by Farooqui et al. [20], who also reported a positive association between anxiety and information need [20,21].

A further explanation is that the audio-visual component may have enhanced patient understanding in a way that written information alone could not fully achieve. Pritchard [3] emphasized that appropriate information is essential for reducing distress in preoperative patients, particularly when time for direct discussion is limited. Similarly, Jlala, French, Foxall, Hardman, and Bedforth [15] argued that multimedia information may help patients understand the perioperative journey more clearly, especially when verbal explanations alone are difficult to retain [15]. Bozkurt et al. [12] and Rostami, Salimi, and Jalalvandi [14] likewise suggested that video-based or electronic educational methods may reduce preoperative anxiety because they present information in a more concrete, understandable, and memorable way. Therefore, the benefit observed in the present study may be attributed not only to the quantity of information provided, but also to the clarity and format in which it was delivered [12,14].

The baseline findings of the current study agree with a wide body of literature showing that preoperative anxiety is common among adults undergoing surgery. Studies from Palestine, India, Pakistan, Nepal, and Ethiopia all reported that a substantial proportion of surgical patients experience significant preoperative anxiety, although the reported prevalence varies across settings and populations [6,9,11,20,21]. This broader pattern is also reflected in the systematic review by Bedaso, Mekonnen, and Duko [5], which found a pooled prevalence of 55.7% among patients undergoing surgery in low- and middle-income countries.

The main outcome of this study, namely the reduction in anxiety after combined written and audio-visual information, is consistent with several intervention studies [12,14,26,27,28,29,30,31,32,33]. Güzel et al. [28] reported that preoperative video-assisted education reduced anxiety and improved comfort in patients undergoing breast cancer surgery. Bozkurt et al. [12] similarly reported significantly lower post-information STAI-S scores in patients who received an information video in addition to written and verbal information. Rostami et al. [14] also found that electronic education significantly decreased preoperative anxiety in patients undergoing laparoscopic cholecystectomy. The present results are also in agreement with more recent studies using immersive technologies [8,13]. Kwon et al. [13] found that virtual reality-based preoperative education significantly reduced both APAIS anxiety and information desire. Chiu et al. [8] likewise reported that a virtual reality intervention reduced preoperative anxiety and stress and improved preparedness in adults undergoing elective surgery. Although the intervention used in the present study was less technologically advanced than virtual reality, the direction of the effect was very similar. This similarity suggests that the key therapeutic element may not be the technology itself, but the ability of the educational approach to make the perioperative process understandable, predictable, and less threatening [8,13].

However, the evidence is not entirely consistent, and some studies have reported no significant reduction in anxiety after video-based interventions [16,34,35,36]. Ouaz et al. [36], in a randomized controlled trial of virtual reality during orthopedic surgery under regional anesthesia, found no significant difference in preoperative anxiety between the intervention and control groups. Likewise, Nami et al. [35] reported that, among older adults undergoing cataract surgery, both an educational video and an operating room visit were associated with higher anxiety scores than standard care. A meta-analysis of virtual reality interventions [34] also found only a small, non-significant overall reduction in preoperative anxiety. These findings suggest that the effectiveness of preoperative educational or immersive interventions may depend on the surgical population, age group, clinical context, and the way patients interpret procedural information. In some groups, especially older adults or populations with specific emotional concerns, greater exposure to procedural details may not reassure patients and may instead intensify anticipatory anxiety [34].

The findings may also be interpreted in light of studies that emphasize the importance of communication and psychosocial support [9,19,37]. Shawahna et al. [9] highlighted the importance of screening preoperative patients for anxiety, particularly those at greater risk, to support more appropriate perioperative care. Salzmann et al. [19] further showed that many anxious patients experience their anxiety as emotionally distressing and would welcome support in coping with it. Marinelli et al. [37] similarly concluded that surgery-related concerns and psychological distress may intensify preoperative anxiety and that better communication is important in helping patients manage these concerns. Thus, the benefit of the intervention in the present study may reflect both educational and supportive effects, because clear information can function not only as instruction but also as reassurance. Combined written and audio-visual education was associated with lower preoperative anxiety and information desire scores after adjustment for baseline scores. These findings suggest that this educational approach may complement routine preoperative education for patients undergoing total knee replacement. By adopting comprehensive multimedia education, institutions may effectively reduce the clinical reliance on pharmacological premedication [5,20] that carries inherent risks of respiratory depression and excessive sedation. Implementing these protocols likely improves postoperative recovery by aligning patient expectations with clinical reality, thereby reducing the incidence of dissatisfaction during the healing phase.

Several limitations should be considered. Although group allocation followed a rolling alternating sequence, the sequence was predictable and was not concealed; therefore, allocation was not random. Because the next assignment could be anticipated, the enrollment or allocation process may have been influenced, creating a risk of selection bias. While our sample size was significant, the nature of a quasi-experimental study and single-center setting means that selection bias cannot be completely excluded. To overcome this limitation, further studies with randomized controlled trials must be conducted in different healthcare facilities to determine whether the benefits of these combined educational modules can be achieved on a broader and more diverse surgical population.

## 5. Conclusions

This study was carried out to determine if a combination of written and audio-visual education can decrease preoperative anxiety in an adults’ patient undergoing total knee replacement surgery, while being able to satisfy the information desire. We found that patients who received this modality of education had significantly lower anxiety levels and needed less additional information than those receiving routine oral instructions.

## Figures and Tables

**Table 1:** Comparison of the sample characteristics between the two groups at baseline (N = 136).

Variable	Experimental Group (n = 68)	Control Group (n = 68)	Test Statistic	*p* Value
Age (years), Mean ± SD	48.35 ± 4.43	48.56 ± 4.50	*t* = 0.27	0.79
Gender, n (%)			χ^2^ = 1.12	0.29
Male	29 (42.6)	23 (33.8)		
Female	39 (57.4)	45 (66.2)		
Marital status, n (%)			χ^2^ = 0.99	0.61
Married	48 (70.6)	43 (63.2)		
Single	5 (7.4)	5 (7.4)		
Divorced/Widowed	15 (22.0)	20 (29.4)		
Educational level, n (%)			χ^2^ = 5.84	0.06
Primary or below	21 (30.9)	33 (48.5)		
Secondary	32 (47.1)	28 (41.2)		
University or above	15 (22.0)	7 (10.3)		
Employment status, n (%)			χ^2^ = 0.08	0.78
Employed	8 (11.8)	7 (10.3)		
Not working *	60 (88.2)	61 (89.7)		
Presence of chronic disease, n (%)			χ^2^ = 3.65	0.06
Yes	54 (79.4)	44 (64.7)		
No	14 (20.6)	24 (35.3)		
History of surgery, n (%)			χ^2^ = 5.01	0.08
None	20 (29.4)	16 (23.5)		
1–2 surgeries	32 (47.1)	44 (64.7)		
>2 surgeries	16 (23.5)	8 (11.8)		
History of anesthesia, n (%)			χ^2^ = 1.59	0.21
Yes	41 (60.3)	48 (70.6)		
No	27 (39.7)	20 (29.4)		
Use of anxiety-related drugs, n (%)			Fisher’s exact = 2.79	0.21
Yes	1 (1.5)	5 (7.4)		
No	67 (98.5)	63 (92.6)		

Note. Values are presented as mean ± standard deviation (SD) or frequency and percentage n (%). Independent samples t-test was used for continuous variables and chi-square test for categorical variables. A two-sided Fisher’s exact test was used to compare anxiety-related drug use because of small expected cell counts. * Not working includes unemployed, housekeeper, and retired participants.

**Table 2:** Distribution of responses to the Amsterdam Preoperative Anxiety and Information Scale (APAIS) items at baseline (before intervention) (N = 136).

Item	Not at All n (%)	A Little n (%)	Moderate n (%)	High n (%)	Extreme n (%)
I am worried about the anesthetic	11 (8.1)	23 (16.9)	45 (33.1)	40 (29.4)	17 (12.5)
The anesthetic is on my mind continually	8 (5.9)	19 (14.0)	51 (37.5)	37 (27.2)	21 (15.4)
I would like to know as much as possible about anesthetics	6 (4.4)	15 (11.0)	44 (32.4)	47 (34.6)	24 (17.6)
I am worried about the procedure	10 (7.4)	19 (14.0)	53 (39.0)	34 (25.0)	20 (14.7)
The procedure is on my mind continually	7 (5.1)	23 (16.9)	52 (38.2)	43 (31.6)	11 (8.1)
I would like to know as much as possible about the procedure	5 (3.7)	10 (7.4)	60 (44.1)	34 (25.0)	27 (19.9)

Note. Values are presented as frequency and percentage n (%).

**Table 3:** Comparison of APAIS anxiety and information desire scores between the control and experimental groups at baseline (before intervention) (N = 136).

Variable	Control Group (n = 68) Mean ± SD	Experimental Group (n = 68) Mean ± SD	*t*	*p*
Anxiety score (Items 1,2,4,5)	12.90 ± 2.26	13.10 ± 1.86	−0.58	0.56
Information desire score (Items 3,6)	6.84 ± 1.43	7.16 ± 1.59	−1.25	0.21

Note. Anxiety score = sum of items 1, 2, 4, and 5. Information desire score = sum of items 3 and 6. Independent samples t-test was used to compare the groups.

**Table 4:** Baseline-adjusted comparison of APAIS anxiety and information desire scores between the control and experimental groups (N = 136).

Variable	Group	Baseline Score (Mean ± SD)	Post-Intervention Score (Mean ± SD)	Adjusted Mean Difference (95% CI)	*p*-Value	Partial η^2^
Anxiety score	Control (n=68)	12.90 ± 2.26	11.49 ± 1.65	−2.25 (−2.84 to −1.66)	<0.001 **	0.30
	Experimental (n=68)	13.10 ± 1.86	9.26 ± 1.84			
Information desire score	Control (n=68)	6.84 ± 1.43	5.46 ± 1.01	−3.14 (−3.42 to −2.87)	<0.001 **	0.78
	Experimental (n=68)	7.16 ± 1.59	2.31 ± 0.58			

Note. Separate ANCOVA models included study group and the corresponding baseline score. Partial η^2^ was calculated from conventional ANCOVA sums of squares. ** *p* < 0.001 (two-tailed).

## Data Availability

The datasets generated during and/or analyzed during the current study are available from the corresponding author on reasonable request.
